# Using harmonicity to facilitate binaural fusion

**DOI:** 10.1121/10.0034883

**Published:** 2025-01-16

**Authors:** Justin M. Aronoff, Jordan Deutsch, Josephine R. LaPapa

**Affiliations:** Speech and Hearing Science Department, University of Illinois at Urbana-Champaign, Champaign, Illinois 61820, USA jaronoff@illinois.edu, jpd4@illinois.edu, jlapapa2@illinois.edu

## Abstract

Harmonicity is an organizing principle in the auditory system, facilitating auditory object formation. The goal of the current study is to determine if harmonicity also facilitates binaural fusion. Participants listened to pairs of two-tone harmonic complex tones that were harmonically or inharmonically related to each other. When the components of two inharmonically related complex tones were divided between the ears, the resulting percept was not binaurally fused. In contrast, when the components of two harmonically related complex tones were divided between the ears, binaural fusion occurred, even absent interaural spectral overlap. This suggests that harmonicity can facilitate binaural fusion.

## 1. Introduction

Binaural fusion reflects the ability to integrate signals presented to the left and right ear into a single auditory image. It is a graded effect, ranging from the percept of a single punctate (i.e., spatially compact) auditory image to two separate auditory images at each ear (Blauert and Lindemann, 1986; Kim and Aronoff, 2021; Suneel *et al.*, 2017). It also can result in the perception of a single pitch when different sounds are presented to each ear, yielding a pitch that is in between the pitch of the sound presented to either ear (Oh and Reiss, 2017; Reiss *et al.*, 2017). Two main factors have been found to play a critical role in binaural fusion. One of these factors is interaural coherence, or the statistical similarity of the signal presented to the two ears, after accounting for interaural time differences. A number of studies have found that increased interaural coherence leads to a perception that is more likely to be binaurally fused and create a punctate rather than a diffuse image (Blauert and Lindemann, 1986; Sayers and Cherry, 1957). A second factor is the extent to which the signal delivered to the two ears stimulates matching locations in the two cochleae, with fusion increasing for stimuli delivered to matched locations (Goupell *et al.*, 2013; Kan *et al.*, 2013; Reiss *et al.*, 2017; Reiss *et al.*, 2018). While these factors have largely been the focus of research on binaural fusion, there is some evidence that they are not the only factors that affect binaural fusion. For example, amplitude modulations can facilitate binaural fusion, potentially preserving binaural fusion for stimuli containing interaurally mismatched frequencies (Oh and Reiss, 2018).

There are a multitude of cues that have been found to facilitate the grouping of spectral components, particularly in the formation of auditory objects (Bregman, 1990). This raises the question of whether cues that foster the grouping of sound components into auditory objects can also be used to group sound components divided across ears to facilitate binaural fusion. One such cue is harmonicity, where sounds that share a common harmonic structure tend to group together into a single auditory object (Hartmann *et al.*, 1990; McDonald and Alain, 2005; Moore *et al.*, 1986). Deviations from harmonicity can cause one auditory object to split into two auditory objects. For example, when one partial in a harmonic complex tone is mistuned by as little as 1%, the mistuned partial is heard as separate from the complex tone (Moore *et al.*, 1986), creating two auditory objects instead of one. This study will investigate the effects of harmonicity on binaural fusion.

## 2. Methods

### 2.1 Participants

Twelve individuals participated in this study (see Table 1). All had audiometric thresholds ≤25 dB HL from 250 Hz to 8 kHz.

**Table 1.** Participant characteristics.

ID	Sex	Age
BHL_333	Female	31
BHL_335	Male	27
BHL_352	Male	30
BHL_360	Male	35
BHL_367	Male	27
BHL_400	Male	47
BHL_452	Male	21
BHL_454	Male	44
BHL_455	Male	20
BHL_457	Male	20
BHL_460	Female	19
BHL_461	Female	20

### 2.2 Stimuli

**Fig. 1.**
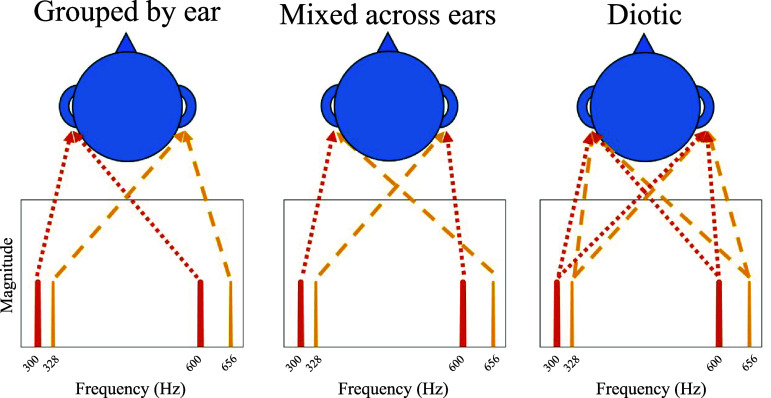
Schematic illustration of the experimental conditions. Harmonically related components are displayed using the same color.

The stimuli were composed of two two-tone harmonic complex tones, each consisting of a fundamental frequency (F0) and its second harmonic. The two complex tones were either harmonically or inharmonically related to each other. The components that made up the two-tone harmonic complex tones were either presented to the same ear or divided between ears (see Fig. 1). One two-tone harmonic complex tone always had a 300 Hz F0. The other two-tone harmonic complex tone had an F0 of 307, 314, 321, 328, 335, 342, 349, or 600 Hz. For the two-tone harmonic complex tone with the 600 Hz F0, the F0 for that complex tone was the second harmonic of the F0 for the other complex tone (i.e., the one with a 300 Hz F0). This meant that all components of both two-tone harmonic complex tones were harmonically related.

As shown in Fig. 1, in the *Grouped by ear* condition, the F0 for one of the two-tone harmonic complex tones and its second harmonic was presented to one ear and the F0 for the other two-tone harmonic complex tone and its second harmonic was presented to the other ear (e.g., 300 and 600 Hz were presented to one ear, and 328 and 656 Hz were presented to the other ear). In the *Mixed across ears* condition, as with the *Grouped by ear* condition, the F0 of one of the two-tone harmonic complex tones was presented to one ear and the F0 of the other two-tone harmonic complex tone was presented to the other ear. However, the second harmonic for each F0 was presented to the opposite ear as the F0 (e.g., 300 and 656 Hz were presented to one ear, and 328 and 600 Hz were presented to the other ear). This means that both ears contained inharmonic tone complexes with harmonically related components occurring in opposite ears. In the *Diotic* condition, both two-tone harmonic complex tones were presented to both ears. Each stimulus had a duration of 1 s with 100 ms raised cosine ramps imposed on the onset and offset of the stimulus.

### 2.3 Procedures

After obtaining informed consent, stimuli were presented to participants who were seated in a double-walled IAC sound booth using Sennheiser HDA 200 headphones and an M-Audio MobilePre external soundcard. The left and right headphones were calibrated separately using an artificial ear, microphone, and preamplifier (Brüel and Kjaer type 4153, 4192, and 2669, respectively). Stimuli were presented at 65 dBA for each ear across all conditions.

**Fig. 2.**
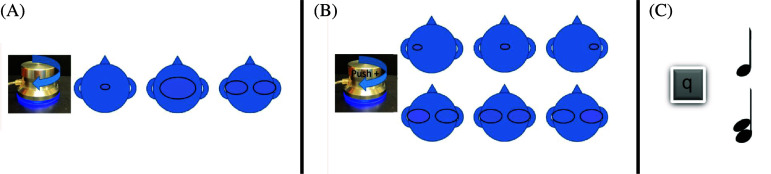
Response interface options. Participants indicated the size and number of auditory images by turning the dial (A). They indicated the laterality of the image if one auditory image was selected or the lateral dominance if two auditory images were selected by pressing down and turning the dial (B). They indicated the number of notes perceived by pressing the “q” key, which alternated between displaying one or two notes (C).

Participants were asked to report on three aspects of the stimuli: the degree to which there was binaural fusion, the lateralization of the percept, and the number of notes that they heard (see Fig. 2). Participants were shown a schematic of a head with an oval superimposed on it. They were provided with a dial (PowerMate, Griffin Technology, Nashville, TN) to manipulate the response image. By turning the dial, they could change the size and number of the oval(s). When they turned the dial clockwise, the oval gradually grew, eventually splitting into two ovals, one near each ear, each of which shrank as the participant continued to turn the dial. This reflected the continuum of binaural fusion found in Blauert and Lindemann (1986) where participants were asked to draw on images of a head to indicate the perceived auditory image. The dial created discreet steps, the number of which were recorded as the “fusion dispersion score.” This score ranged from 0 to 17. A score of 10 or greater indicated two auditory images and was reflected as two ovals in the interface.

By pushing down and turning the dial, participants indicated the lateralization of the percept. If they indicated there was one auditory image, pushing down and turning the dial to the left moved the oval to the left and pushing down and turning the dial to the right moved the oval to the right. If they indicated there were two auditory images, pushing down and turning the dial allowed participants to indicate which image was more dominant, with the less dominant oval color changing to more closely match the color of the head in the image and the more dominant oval color changing to a pinker hue. The discreet steps of the dial corresponded to the “lateralization score,” ranging from −10 (left) to 10 (right). 0 indicated a centered image.

Participants also indicated the number of notes that they perceived by pressing the “q” key on the keyboard. Pressing it once placed an image of a note above the head image. Pressing it again placed an image of two notes in that same location. Continuing to press the “q” key alternated between these two options. After each new stimulus was presented, the image of the note(s) was removed from the screen. Participants could not record their response until they had pressed the “q” key at least once (i.e., until there was an image of one or two notes). Participants could repeat a given stimulus with a button on the interface if they desired. After participants indicated the number of images, image lateralization, and number of perceived notes, they pressed the space bar to record their response. This caused the next stimulus to be played. Participants completed two blocks, with each block containing all 24 stimuli (8 F0 combinations × 3 conditions).

### 2.4 Analysis

Robust statistics were used to analyze the data given the ample evidence from simulations that such statistics typically yield more accurate results and greater power than traditional methods (Erceg-Hurn and Mirosevich, 2008; Wilcox, 1998; Wilcox and Keselman, 2003). Bootstrap analyses were used to avoid the detrimental effects of non-normality on traditional analyses and to model the dependency in the data. For bootstrap analyses, the distributions being analyzed were generated by random sampling with replacement from the original dataset. For the number of notes perceived (interval data), 20% trimmed means were used to minimize the effects of outliers and skewed distributions in the group analyses. These reflect the mean of the central 60% of the data. Familywise error was corrected using Rom's method (Rom, 1990), which is a sequential rejection method that minimizes both type I and type II errors.

Bootstrap correlations were used, controlling for dependencies within a participant's data by creating bootstrap samples by randomly selecting a participant with replacement and including their entire dataset. This was repeated for the same number of participants as there were in the original dataset (i.e., 12 participants). This will be collectively referred to as a bootstrap sample. A Spearman rank correlation or a Pearson correlation, depending on if interval or ordinal data were being analyzed, was calculated for each bootstrap sample. This process was repeated 599 times per group, and the confidence interval for the correlations was determined.

## 3. Results

The lateralization score for the stimuli did not significantly differ from 0 (perceptually centered) for any stimulus or condition. As such, lateralization scores were not incorporated in any of the following analyses.

### 3.1 The effect of condition and stimulus on binaural fusion

**Fig. 3.**
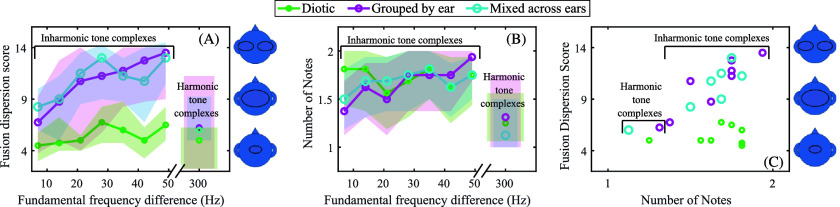
Results indicating that harmonicity improved binaural fusion. (A) The median fusion dispersion score for each condition as a function of the fundamental frequency difference for the two two-tone harmonic complex tones in each stimulus. Shaded areas represent the 95% confidence interval of the median. (B) The 20% trimmed mean of the number of notes perceived for each condition as a function of the fundamental frequency difference for the two two-tone harmonic complex tones in each stimulus. Shaded areas represent the 95% confidence interval of the 20% trimmed mean. (C) The relationship between the 20% trimmed mean of the number of perceived notes and the median fusion dispersion score for each condition.

The relationship between the F0 separation of the two-tone harmonic complex tones and binaural fusion was examined. As shown in Fig. 3(A), when the two two-tone harmonic complex tones in the stimuli were inharmonically related, fusion dispersion scores increased with increasing differences between the F0s of the two-tone harmonic complex tones. This was confirmed with bootstrap Spearman rank correlations for each condition with familywise error corrected using Rom's method. There was a significant correlation for the *Diotic* condition (r = 0.22; 95% confidence interval: 0.06 to 0.41), *Grouped by ear* condition (r = 0.38; 95% confidence interval: 0.23 to 0.51), and *Mixed across ears* condition (r = 0.33, 95% confidence interval: 0.11 to 0.51). This indicated that increased deviations from matched F0s increased the binaural fusion dispersion score.

As shown in Fig. 3(A), binaural fusion dispersion scores were notably lower (more fused) with the *Diotic* condition than the other conditions when the two two-tone harmonic complex tones were inharmonically related. To examine this, Friedman rank sum tests followed by Wilcoxon signed-rank tests were conducted, comparing the binaural fusion dispersion score for the three conditions for either the stimulus with the 49 Hz F0 difference or when pooled across all conditions with stimuli with inharmonically related F0s. Familywise error was controlled using Rom's method. There was a significant effect of condition for both analyses (χ^2^(2) = 7.58; p < 0.05 for the stimuli with the 49 Hz F0 difference; χ^2^(2) = 12.7; p < 0.01 when pooled across all inharmonic tone complexes). When analyzing only the 49 Hz F0 difference, fusion dispersion scores were significantly lower for the *Diotic* condition than for either the *Grouped by ear* condition (z = 2.51, p < 0.05; r = 0.72) or the *Mixed across ears* condition (z = 2.36, p < 0.05; r = 0.68). However, there was no significant difference between the *Grouped by ear* and the *Mixed across ears* condition (z = −0.78, p > 0.05; r = −0.22). When pooling across all inharmonic tone complexes, fusion dispersion scores were significantly lower for the *Diotic* condition than for either the *Grouped by ear* condition (z = 2.80, p < 0.05; r = 0.81) or the *Mixed across ears* condition (z = 2.85, p < 0.05; r = 0.82). However, there was no significant difference between the *Grouped by ear* and the *Mixed across ears* condition (z = −0.93, *p* > 0.05; r = 0.27).

To determine if harmonicity increased binaural fusion, the binaural fusion dispersion scores for the stimuli containing the two-tone harmonic complex tones with a 300 and 349 Hz F0 (i.e., when the difference in the F0s of the two two-tone harmonic complex tones in the stimulus was greatest without being harmonically related) were compared to those for the stimuli containing the two-tone harmonic complex tones with a 300 and 600 Hz F0 (where all components of both two-tone harmonic complex tones were harmonically related) for each condition. As shown in Fig. 3(A), there was a notable decrease in fusion dispersion scores (i.e., more fusion) when the harmonic tone complex was presented. Wilcoxon signed-rank test for each condition with familywise error corrected using Rom's method indicated that the decrease was significant for all conditions (all *p* values < 0.05; median fusion dispersion difference for *Diotic* condition: 1.25, r = 0.77; median fusion dispersion difference for *Grouped by ear* condition: 5.75, r = 0.75; median fusion dispersion difference for *Mixed across ears* condition: 4, r = 0.70).

### 3.2 The effect of condition and stimulus on the number of perceived notes

It was anticipated that, as the difference of the F0s of the two-tone harmonic complex tones increased, participants would be more likely to hear two notes when the F0s were not harmonically related. Figure 3(B) suggests that this was the case. This was analyzed with bootstrap Pearson correlations for each condition with familywise error corrected using Rom's method. There was a significant correlation for the *Grouped by ear* condition (r = 0.26; 98.3% confidence interval: 0.01 to 0.50), but not for the *Diotic* condition (r = −0.03; 98.3% confidence interval: −0.21 to 0.11) or for the *Mixed across ears* condition (r = 0.12; 98.3% confidence interval: −0.07 to 0.26). This indicates that increasing the difference between F0s when they were not harmonically related only had a demonstrably systematic effect for the *Grouped by ear* condition.

The lack of a significant correlation for the other conditions could indicate that harmonicity did not decrease the number of perceived notes or that even a 7 Hz F0 difference was sufficient to increase the number of perceived notes. To examine this, the number of perceived notes for the *Diotic* and the *Mixed across ears* conditions for the stimuli containing the two-tone harmonic complex tones with either a 7 Hz or a 49 Hz F0 difference were compared with those with a 300 Hz F0 difference (i.e., when all components were harmonically related) using pairwise comparisons with 20% trimmed means with familywise error corrected using Rom's method. This indicated that participants were not significantly more likely to indicate they perceived more notes with the inharmonically related two-tone complex tones compared to the harmonically related two-tone complex tones for the *Diotic* condition (20% trimmed mean difference: 0.31 notes; *p* > 0.025 based on the Rom's corrected alpha of 0.025) or for the *Mixed across ears* condition (20% trimmed mean difference: 0.19; *p* > 0.025) when the F0s for the two two-tone harmonic complex tones differed by only 7 Hz. In contrast, when the F0s for the two two-tone harmonic complex tones differed by 49 Hz, there was a notable increase in the number of perceived notes for the inharmonically related two-tone complex tones compared to the harmonically related two-tone complex tone (*p* values < 0.05 for both; 20% trimmed mean difference for *Diotic* condition: 0.31 notes; 20% trimmed mean difference for *Mixed across ears* condition: 0.44 notes).

### 3.3 The relationship between binaural fusion and the number of perceived notes

To determine if fusion dispersion scores were related to the number of perceived notes, the relationship between these two measures was analyzed. As highlighted by Fig. 3(C), fusion dispersion scores were related to the number of perceived notes when the stimuli were dichotic. Bootstrap Spearman rank correlations were conducted comparing binaural fusion dispersion to the number of notes perceived for the inharmonically related two-tone harmonic complex tones. Familywise error was corrected using Rom's method, resulting in an alpha of 0.025. There was a significant correlation for the *Grouped by ear* condition (r = 0.41, 98.75% confidence interval: 0.004 to 0.70) and the *Mixed across ears* condition (r = 0.34; 98.75% confidence interval: 0.06 to 0.63), indicating a relationship between perceiving two tones and lower binaural fusion dispersion when the stimuli were dichotic. In contrast, the correlation for the *Diotic* condition was not significant (r = −0.04, 98.75% confidence interval: −0.41 to 0.35), suggesting that participants were not conflating the perception of two notes with the perception of two auditory images, one at each ear.

## 4. Discussion

This study investigated the use of harmonicity for facilitating binaural fusion. The results indicate that stimuli were perceived as having a lower binaural fusion dispersion score in the presence of harmonicity, despite the stimulus with the harmonically related two-tone harmonic complex tones containing the largest F0 difference. This decrease in binaural fusion dispersion occurred regardless of whether each ear received components that were harmonically related to a perceivable pitch or not. It is not clear if the resulting binaural fusion with harmonically related tone complexes was obligatory or if participants could have chosen not to binaurally fuse the signal (Bregman *et al.*, 2001; van Noorden, 1975), and it is possible that the effect of harmonicity would be strengthened with the use of additional harmonically related tones in each harmonic tone complex, given that an increased number of harmonic components can improve pitch identification (Houtsma and Smurzynski, 1990).

For the *Grouped by ear* and *Mixed across ears* conditions, there was a significant relationship such that an increased likelihood of perceiving two notes corresponded to an increased fusion dispersion score. This suggests that both aspects of fusion that have been discussed in the literature (i.e., perceiving a single auditory image and a single sound or pitch; see Reiss and Goupell, 2024) may be two aspects of the same binaural fusion phenomenon.

The decrease in the number of perceived notes for the *Diotic* condition compared to when the fundamental frequencies differed by 7 Hz (approximately 2.5% of the harmonics for the other F0) is consistent with previous results that have shown that increasing the mistuning of one partial by 1%–3% results in it separating from the remaining partials (Moore *et al.*, 1986). This was notably different for the *Grouped by ear* condition, where the F0 difference for the inharmonically related two-tone complex tones was significantly correlated with the number of perceived notes. Similarly, for the *Mixed across ear* condition, there was not a significant increase in the number of perceived notes for a 7 Hz F0 difference compared to when all components were harmonically related. This suggests a greater challenge in perceiving mistuned harmonics across ears, consistent with results from Oh *et al.* (2022), although it could also reflect the availability of amplitude modulation-based cues caused by the inharmonic relationship between the two tones in the complex tone in the *Diotic* condition.

One possible explanation for the relationship between harmonicity and binaural fusion could be that participants were conflating the different response options, indicating that they heard one auditory image (low fusion dispersion score) based on the number of notes they heard. However, unlike with the other conditions, for the *Diotic* condition, participants indicated a range of the number of perceived notes but still indicated low fusion dispersion scores for all stimuli.

While there was a large effect of dividing inharmonically related two-tone harmonic complex tones across ears in terms of binaural fusion, there was little effect of whether the harmonic components were grouped within or across ears. This may indicate that harmonicity cannot facilitate separating signals across ears, which stands in contrast to previous work suggesting that harmonicity can improve signal detection in noise for diotic stimuli (Rajappa *et al.*, 2023).

## 5. Conclusion

The results of this study indicated that harmonicity can help foster binaural fusion. However, grouping harmonically related components within each ear does not appear to additionally affect binaural fusion. The effects of harmonicity on binaural fusion raise the possibility that there may be other auditory grouping cues that can lead to a more binaurally fused and punctate image.

## Data Availability

The data that support the findings of this study are available from the corresponding author upon reasonable request.
